# The Responsibility of Veterinarians to Address Companion Animal Obesity

**DOI:** 10.3390/ani8090143

**Published:** 2018-08-21

**Authors:** Barry S. Kipperman, Alexander J. German

**Affiliations:** 1 Department of Veterinary Medicine and Surgery, University of Missouri College of Veterinary Medicine, Columbia, MO 65211, USA; 2 Institute of Ageing & Chronic Disease, University of Liverpool, Neston CH60 5SZ, UK; ajgerman@liverpool.ac.uk; 3 Institute of Veterinary Science, University of Liverpool, Neston CH60 5SZ, UK

**Keywords:** ethics, obesity, communication, advocacy, one health

## Abstract

**Simple Summary:**

Obesity is a disease of rapidly increasing prevalence in dogs and cats, with significant and often lifelong implications for animal welfare. Veterinarians are expected and mandated to protect animal health and welfare, and provide informed consent. We provide an overview of the causes, risk factors, and consequences of pet obesity; evidence regarding veterinarian compliance in diagnosing and discussing obesity in small animal practice; and outline recommendations to prevent and address overweight and obesity in companion animals. We argue that small-animal veterinarians are not meeting their ethical and professional obligation to speak up about obesity, which is a One-Health problem.

**Abstract:**

Obesity is a modern-day epidemic in both people and companion animals. A summary of the current research on the causes, risk factors, consequences, and implications of overweight and obesity, and the compliance of small-animal practitioners in recognizing and addressing pet obesity, is provided. Ethical and animal welfare concerns are raised regarding these findings. We argue that a patient advocacy posture compels the veterinary profession to confront this issue more reliably. Evidence is presented to support obesity as a One-Health problem, and discrete and practical recommendations for preventing and addressing companion animal obesity are proposed. The One-Health perspective encourages coordinated action by veterinary healthcare professionals in order to address overweight and obesity in companion animals as a public health concern.

## 1. Introduction

Obesity is a condition where excess body fat has developed to the point that health is adversely affected [1,2]. Obesity is a modern-day epidemic; the results of a survey between 2015 and 2016 in the United States found that 40% of over 27,000 adult people had obesity [3]. Unfortunately, there is no universally accepted definition of canine and feline obesity. Different publications define obesity as being more than 15% [4,5], 20% [6], and 30% above the ideal weight [7]. The American Veterinary Medical Association (AVMA) has recently endorsed a recommendation for a uniform definition, whereby obesity is present when a dog or cat is >30% above its ideal weight [8,9]. Body condition scoring (BCS) is the most commonly used method to document adiposity in companion animals, and 30% above the ideal weight corresponds to a score of 8/9 using the preferred nine-point system; this is equivalent to a score of 4.5 using the five-point system with half units [10,11,12,13]. Dogs and cats are considered overweight when their weight is more than 10–20% above their ideal weight, and their corresponding BCS is either 6/9 or 7/9 (3.5/5 and 4/5) [14]. Using BCS, 54% of dogs and 59% of cats in the United States are estimated to be overweight or obese, while a recent study in the United Kingdom classified 65% of adult dogs and 37% of juvenile dogs as overweight or obese [15,16].

Many veterinarians are concerned that the prevalence of pet overweight and obesity is increasing, and this is supported by recent data, albeit not from peer-reviewed publications. For example, over a 10-year period, Banfield^®^ pet hospitals reported a 169% and 158% increase in the prevalence of overweight cats and dogs, respectively [17], while the prevalence of obesity in a five-year period in the United Kingdom increased by 37% in dogs and 90% in cats [6]. Alternatively, these changes may reflect an increased recognition of overweight and obese pets by veterinary practitioners, rather than an actual change in the disease prevalence.

## 2. Causes of Companion Animal Obesity

It seems logical that the rise in the prevalence of obesity in humans is reflected in the companion dogs and cats with whom lifestyles are shared [18]. Studies suggest that the owners of overweight and obese dogs and cats use food as a pivotal means of interaction, communication, and bonding with their animal companions, that is, as a vehicle to express affection and love [19,20,21]. One report concluded that “owners of obese dogs tend to interpret their dog’s every need as a request for food” [22]. Consequently, it is very conceivable that dogs and cats condition or manipulate their owners into overfeeding them by appearing in the kitchen and vocalizing whenever meals are being prepared or by lingering near the table when meals are being consumed [14,18]. Once these behaviors become regularly reinforced via food provision, extinction is very difficult. Neutering has been documented as a risk factor towards being overweight or obese in dogs and cats compared to intact pets [23,24,25,26,27,28].

## 3. Risk Factors and Consequences of Companion Animal Obesity

The Obesity Medicine Association’s definition of obesity is “a chronic, relapsing, multifactorial, … disease, wherein an increase in body fat …, results in adverse metabolic, biomechanical, and psychosocial health consequences” [29]. Overweight dogs are more likely to be diagnosed with hyperadrenocorticism and urinary tract disease, and obese dogs are more likely to be diagnosed with diabetes mellitus, respiratory dysfunction, pancreatitis, and neoplasia [23,30,31]. Overweight and obese dogs are at increased risk of developing hypothyroidism and orthopedic disorders (osteoarthritis and cruciate ligament rupture) [23,32]. Overweight cats are at increased risk for developing urinary tract disease, diabetes mellitus, respiratory disease, and orthopedic diseases; obese cats are at increased risk for diabetes mellitus and neoplasia; and overweight and obese cats are at higher risk of dermatological conditions, oral conditions, hypertension, and diarrhea [33,34,35,36]. It should be noted that distinguishing cause vs. effect for these risk factors can be challenging. As an example, a dog with significant orthopedic disease may be less likely to ambulate and exercise, which increases its risk of overweight or obesity. But, overweight may also cause orthopedic disorders.

Health related quality of life is also worse in dogs with obesity compared with dogs at ideal weight, and obese dogs also have increased risk associated with anesthesia [14,37]. Consequently, the average lifespan is shorter in overweight dogs by about two years [38,39]. Surprisingly, a recent study found that the survival times in overweight and obese cats were not reduced [40]. Overweight and obesity also impose a significant financial burden on pet owners, with a recent report revealing that the owners of overweight dogs spent 17% more on health care and 25% more on medications compared with the owners of healthy-weight dogs [17]. In the same report, the owners of overweight cats spent 36% more on diagnostic procedures, compared with the owners of healthy-weight cats [17].

## 4. The Role of Veterinarians

According to the Veterinary Oath, veterinarians have an obligation to protect animal health and welfare, promote public health, and prevent and relieve animal suffering [41]. An AVMA policy statement on informed consent states, “Informed consent better protects the public by ensuring that veterinarians provide sufficient information in a manner so that clients may reach appropriate decisions regarding the care of their animals. Veterinarians, to the best of their ability, should inform the client … of the … risk assessment and prognosis …” [42]. As overweight and obese states impact longevity in dogs and quality of life in dogs and cats, and the condition can be reversed via dietary and lifestyle modifications, these codes of conduct suggest that veterinarians should address the issue of pet obesity each time it is recognized.

## 5. Compliance with Diagnosing and Discussing Obesity

Our collective experience of over 50 years in referral practice as internal medicine specialists has been that general practitioners seldom record the recognition or discussion of overweight or obesity with pet owners. A retrospective study of the primary care veterinary records of 148 dogs in the United Kingdom over an approximately 12-month interval found that only 70% of the medical record entries documented the patient body weight [43]. Only 29% of these records contained a qualitative assessment of the body condition, only 15% of records documented obesity, and a body condition score was documented in only one record for one dog. A survey-based study of 48 small-animal practices and 2661 dogs in Australia discovered that the practitioners categorized 41% of the patients as overweight or obese, but often did not inform the owners when a dog was overweight [44]. Another retrospective report of 74 general practices in the United Kingdom, involving more than 49,000 dog visits, documented that only 1.4% of all of the record entries contained words pertaining to ‘overweight’ or ‘obese’ [45]. These findings demonstrate that small-animal veterinarians are not recognizing and discussing pet overweight and obesity with their clients.

## 6. Ethical Concerns

Professional ethics poses questions such as, “What *is* the veterinary profession doing about the issue of obesity?”, and “What *should* the profession be doing about obesity?”. We contend that there is a significant and highly concerning gap between the answers to these questions. A logical conclusion is that small- animal practitioners may be reluctant to confront the issue of pet obesity for fear of alienating or jeopardizing their relationship with the client. Supporting this hypothesis, a qualitative study of 15 general practitioners in the Netherlands indicated that the most common obstacles in discussing obesity with pet owners were lack of time (66% of veterinarians) and a fear of losing the client (50% of respondents) [46]. The results of another report substantiate that time constraints and concerns of offending the client were suggested causes for the practitioners’ failure to discuss pet obesity [47]. Other contributing factors to poor compliance among veterinarians in discussing obesity may include the habituation of or desensitization to recognizing obesity given its prevalence, a normalization of obesity (analogous to the issue of brachycephaly in which clients and practitioners report that noisy breathing is “normal for the breed”) [48], prioritization of the emotional component of the human–animal bond over the patient’s physical health, or a belief that client compliance in addressing their pet’s obese state would fail, regardless of professional concern, recognition, or guidance.

One of the fundamental questions facing veterinarians is whether their primary allegiance is to the animal or to the pet owner. In a recent study, only 20% of small-animal veterinarians indicated that other practitioners prioritize the patients’ interests, and only 50% of practitioners characterized their own behavior as prioritizing their patients [49]. One barrier discouraging the animal advocacy position is economic; the veterinarian is dependent on the pet owner who pays for the veterinary services [50]. These findings raise questions regarding whether the majority of small-animal veterinarians see their professional role as primarily advocates for animals.

## 7. Reasons for Speaking Up about Obesity

When animal welfare scientists make assessments regarding which issues affecting welfare warrant the allocation of time, labor, and resources, the following four criteria are considered: number of animals affected, duration of affect, impact on quality of life, and reversibility. If we apply these standards to the issue of pet obesity, we can readily surmise that about 80 million dogs and cats in the United States are overweight or obese [51]. The great majority of these animals will remain obese for a significant portion of, or for the remainder of their lifespan; obesity causes significant impairments to welfare, and it has been confirmed that dogs that successfully lose weight have a significantly improved quality of life [37]. Furthermore, the results of a prospective longitudinal study of 39 Labrador Retrievers, followed from six years of age until death, concluded that the lifelong maintenance of lean body mass and a reduced accumulation of body fat were associated with a longer than average lifespan [52]. Based on these facts, preventing pet obesity provides veterinarians with a significant opportunity to protect animal welfare.

## 8. Obesity Is a One-Health Problem

A consensus statement by the One Health Committee of the World Small Animal Veterinary Association in association with the U.S. Centers for Disease Control and Prevention notes the following [53]:

1- Obesity is a disease and should be referred to as such.
2- Obesity is a disease of increasing prevalence in human and companion animal populations.
3- Obesity is associated with numerous comorbidities in people and pets.
4- Human and veterinary healthcare providers find it difficult to discuss obesity with clients.
5- The prevention of obesity should be a major priority for the human and veterinary health care professions.
6- The veterinarian has a role in improving the health of pets, as well as the pet owners, as caregivers.

Studies have documented an association between lower household incomes and a reduced education of the head of household, with human overweight and obesity [54,55]. A number of studies have identified a positive relationship between obesity in dogs and their owners [22,56,57,58]. The owners of obese dogs may transfer their personal eating habits and diminished interest in their own health, onto their dogs [22], and the owners of overweight or obese dogs and cats underestimate the body condition of their animals [20,59,60,61]. These findings make addressing obesity by the veterinarian more challenging, yet morally compelling. Motivating humans to improve the body condition of their animal companions could be the gateway to recognition and to taking action in confronting their own overweight or obese state. There is some precedent to support this idea, as it has been documented that overweight owners and their overweight companion animals can lose weight together, and that companion dogs can serve as social support during the weight loss period [62]. Conversely, inadequacies in the physical or mental health of the human caregiver may have detrimental ramifications for companion animals in terms of reduced compliance with diets and medications prescribed to the animal dependent [63].

## 9. Recommendations for Addressing Companion Animal Obesity

Overweight and obesity in companion animals impairs quality of life, shortens the lifespan in dogs, and once established, is difficult to reverse. With a rising prevalence, it is a major welfare concern; yet, there is cause for optimism that an increased willingness and motivation by small-animal veterinarians to address weight gain can succeed in achieving weight loss. Approximately two-thirds of pet owners who agreed with their veterinarian’s assessment that their dog was overweight were motivated to take action to reduce weight [47]. The strength of that motivation appeared to correlate with the information provided during the consultation. This study concluded that a combination of a discussion of weight, health risks, and the provision of advice on how to best achieve weight loss via dietary change, was most likely to motivate clients to take appropriate action.

Veterinarians and veterinary paraprofessionals should record the patient’s body weight and BCS at each visit, and the surveillance of previous weights (if available) should be reviewed to assess trends [6]. We suggest discussion and documentation of increasing or declining patient weights of 5% or more, as in keeping with our duty to provide informed consent. As making the diagnosis of overweight and obesity is straightforward, and the health and welfare consequences of the disease are well known, the veterinary profession needs to take a proactive stance regarding this problem. Such an approach should prioritize prevention rather than resolution, aided by lifelong body weight monitoring.

We recommend that veterinarians weigh growing dogs and cats monthly until they reach skeletal maturity, and monitor the rate of growth using growth charts [64,65]. This will improve the likelihood of the dog or cat reaching skeletal maturity at an optimal body weight, confirmed by assessing the BCS. The animal’s early adult ideal bodyweight can be recorded in the clinical notes and used as a reference during adult life. From skeletal maturity until the senior life stage, we recommend that dogs and cats be weighed at least every six months, and then at least every three months in senior patients [6]. Such a program would enable small increases in bodyweight to be identified early, before the animal is formally considered to be overweight, thus corrective measures could be implemented. In addition, older patients losing weight could be identified at an earlier stage of illness. If veterinary practices are not able to offer such frequent weight checks, a weight and body condition score should be obtained annually, at a minimum.

We advise that all patients in weight loss programs be weighed at least every month. If weight loss is documented, positive reinforcement and praise should be provided to encourage continued compliance. If there has not been satisfactory weight loss, further information should be gathered to identify the reasons. For small dogs and cats, owners can acquire a suitable digital scale, so that the pet owner is empowered to obtain the weights at home, and call in with monthly reports. This also reduces the stress of transport to the hospital, especially for cats. The success of weight loss initiatives requires a team approach including pet owners, paraprofessional staff and practitioners. This strategy can be accomplished without stigmatizing, shaming, or alienating the pet owner.

Lack of time is a commonly cited reason for failing to discuss overweight or obesity [46,47]; this raises the question of whether clients and pets would be better served by extending the time allotted for select appointments so as to ensure that the important issue of obesity can be properly addressed. This may increase the care costs to clients in the short term via longer consultations, but those costs would be offset by the long-term benefits of reduced expenditures for the testing and treatment of overweight- and obesity-associated conditions. The greatest benefit may be unmeasurable, that being improving the quality of life of their animal companion via weight loss.

Some may argue that the primary role of the veterinarian is to maximize the human–animal bond, and that focusing on modifying dietary regimens for overweight or obese pets infringes on the bond and causes more emotional harm to the human and animal than the potential benefits are worth. We believe that the veterinarian should encourage the client to not only diminish caloric intake as the pivotal means of resolving obesity, but to also divert the energy and positive intentions previously associated with overfeeding into other endeavors that may be more beneficial to the emotional or physical health of both the animal and human, such as playing with toys or other animal companions, walking, or jogging [62]. Pursuing such a goal respects the importance of preserving the human–animal bond while ensuring that practical and feasible tools to mitigate obesity are provided.

## 10. Conclusions

Veterinarians are not adequately addressing the pet obesity epidemic, and are thereby abdicating their role as advocates for animals. Preemptive monitoring to prevent obesity from developing is strongly encouraged. A combination of informing pet owners that their pet is or may become overweight, discussing the health and welfare implications of obesity, and providing information on how to achieve weight loss, is the most effective mechanism for motivating pet owners to improve the health and welfare of their overweight or obese animal companions. The One-Health perspective encourages coordinated action by human and veterinary healthcare professionals to address obesity in people and companion animals as a public health concern [66].
